# Development of an AAV DNA-based synthetic vector for the potential gene therapy of hemophilia in children

**DOI:** 10.3389/fmicb.2022.1033615

**Published:** 2022-10-06

**Authors:** Jakob Shoti, Keyun Qing, Arun Srivastava

**Affiliations:** ^1^Division of Cellular and Molecular Therapy, Department of Pediatrics, University of Florida College of Medicine, Gainesville, FL, United States; ^2^Powell Gene Therapy Center, University of Florida College of Medicine, Gainesville, FL, United States; ^3^Department of Molecular Genetics and Microbiology, University of Florida College of Medicine, Gainesville, FL, United States

**Keywords:** AAV, inverted terminal repeats, AAV vectors, gene therapy, hemophilia

## Abstract

Recombinant AAV serotype vectors and their variants have been or are currently being used for gene therapy for hemophilia in several phase I/II/III clinical trials in humans. However, none of these trials have included children with hemophilia since the traditional liver-directed AAV gene therapy will not work in these patients because of the following reasons: (i) Up until age 10–12, the liver is still growing and dividing, and with every cell division, the AAV vector genomes will be diluted out due to their episomal nature; and (ii) Repeated gene delivery will be needed, but repeat dosing, even with an ideal AAV vector is not an option because of pre-existing antibodies to AAV vectors following the first administration. Here we describe the development of an optimized human Factor IX (hF.IX) gene expression cassette under the control of a human liver-specific transthyretin promoter covalently flanked by AAV inverted terminal repeats (ITRs) with no open ends (optNE-TTR-hF.IX), which mediated ~sixfold higher hF.IX levels than that from a linear TTR-hF.IX DNA construct in human hepatoma cells up to four-weeks post-transfection. In future studies, encapsidation of the optNE-TTR-hF.IX DNA in liver-targeted synthetic liposomes, may provide a viable approach for the potential gene therapy for hemophilia in children.

## Introduction

Gene therapy with recombinant adeno-associated virus (AAV) vectors has made substantial strides in the last decade with numerous clinical trials showing promise in treating various mono-genetic disorders, including hemophilia. Hemophilia is set of blood clotting disorders where the afflicted individual is unable to efficiently form clots to relieve bleeding or hemorrhaging. This clotting disruption leads to multiple phenotypic manifestations including excessive bleeding from minor cuts, easy bruising, bleeding in joints, and easy bleeding in the brain that could contribute to intracranial hemorrhaging (Rogers and Herzog, 2015). These disorders are genetically inherited in an X-recessive manner and are the result of aberrant genetic alterations to the co-factors and/or zymogens of the coagulation cascade. Hemophilia A is caused by a disruption of the F.VIII gene and aberrant protein product, while Hemophilia B is associated with FIX. Hemophilia A is a more prevalent disorder than Hemophilia B (1:5,000 to 1:25,000) due to F.VIII’s much larger gene size than that of F.IX (Rogers and Herzog, 2015). Considering the size of the genes and the multitude of possible genetic alterations that could yield an afflicted phenotype, there is a wide spectrum of phenotypic severity in hemophilia patients. Phenotypically normal patients have factor levels between 50 and 150% of normal blood clotting factor, meaning a patient could have half the corresponding factor and still not show phenotypic symptoms. Mild severity (25% of hemophilia patients) involves factor levels <5% to <40% with patients exhibiting increased bleeding following injury, trauma, or surgery (Makris et al., 2018). Roughly 15% of hemophilia patients have moderate severity with 1 to 5% factor levels and suffer from increased bleeding following minor injuries with rare spontaneous bleeding events. Those with severe hemophilia make up 60% of hemophilia patients and have factor levels <1%. These patients can suffer from excessive bleeding after minor injuries, increased spontaneous bleeding events, and bleeding into joints and muscles (Makris et al., 2018).

Present treatment for hemophilia consists of routine blood infusions of the respective factor. This prophylactic enzyme replacement therapy has proven very effective in phenotypic remediation with a significant reduction in bleeding events post-infusion (Manco-Johnson et al., 2007). Considering this, these enzymes have a relatively short half-life, ~12 h for FVIII and 18–24 h for F.IX, and infusions have to be administered regularly, 2× weekly and once bi-weekly, respectively, (Graf, 2018). The yearly cost of these infusions can range between $140,000 and $1 M a year depending on severity resulting in medical expenses roughly nine times that of the average patient (Machin et al., 2018). At the physiological level there is the risk of thrombotic episodes with the fluctuating factor serum levels between administrations especially during the first month of treatment. Antibody formation against these factors have been observed in patients with null protein synthesis (Acharya, 2016). Additionally, the routine administrations can be cumbersome and trying for individuals and their families. This is particularly so for children with hemophilia. Additional limitations for AAV vector-mediated gene therapy of hemophilia in children include the rapidly dividing nature of liver up until the age of 10–12 years, leading to loss of AAV vector genomes due to their episomal forms, and the inability of repeat-dosing of AAV vectors due to humoral immunity because of neutralizing antibodies (NAbs) to AAV-capsid proteins. Repeat-dosing with AAV vectors, even in adult patients, is also currently not possible, given the development of NAbs to AAV capsid proteins following the first dosing, although several approaches are currently being developed to overcome this limitation.

To overcome these limitations of AAV vectors for gene therapy of hemophilia in children, we hypothesized that the use of a synthetic AAV vector, devoid of AAV capsid proteins, and capable of repeat-dosing, could be developed. To this end, we further improved a novel no-end (NE) AAV DNA, a previously described hybrid genome that consists of a gene cassette covalently flanked by AAV inverted terminal repeats (ITRs) with no open ends (Nahreini et al., 1992). This linear, NE-DNA is exonuclease-resistant, and can be used to express a transgene following delivery into mammalian cells, yet the stability of NE-DNA and kinetics of transgene expression have not been studied in detail. In the present study, we updated the manufacturing protocol to a synthetic DNA method and assessed various NE-DNA designs based on ITR truncations in immortalized hepatocyte cell lines. This analysis led to an optimized NE-DNA (optNE-DNA) design that was capable of increased DNA and transgene expression longevity in both reporter and therapeutic gene applications. In future studies, encapsidation of the optNE-DNA containing hF.IX or hF.VIII genes in liver-targeted synthetic liposomes, may provide a viable approach for the potential gene therapy for hemophilia B and hemophilia A, respectively, in children.

## Materials and methods

### Cell lines and cultures

Human hepatocellular carcinoma cells, Huh7 and HepG2, were purchased from ATCC (Old Town Manassas, VA, United States) and were maintained in Dulbecco’s-modified Eagle medium (DMEM; Gibco, Waltham, MA, United States) supplemented with 10% fetal bovine serum (FBS; Gibco, Waltham, MA, United States) and 1% penicillin/streptomycin (Invitrogen, Carlsbad, CA, United States) as described previously (Yin et al., 2021). Cell cultures were grown at 37°C and 5% CO_2_. Cells were passaged using trypsin-Versene mixture (Lonza, Walkersville, MD, United States), and resuspended in complete DMEM.

### Plasmids

All DNA constructs used were derived from plasmid DNA sequences either directly through digestion or following PCR amplification. The pSub201 plasmid was utilized for ITR digestion (Samulski et al., 1987). Plasmids used were AAV packaging plasmids with GOI flanked by ITRs. These plasmids included chicken-beta actin promoter (CBA)-driven enhanced green fluorescent protein (pCBA-EGFP), alpha-feto-protein promoter driven EGFP (pAFP-EGFP), transthyretin promoter (TTR) driven EGFP (pTTR-EGFP), and TTR promoter driven human Factor IX-Padua (pTTR-FIX).

### No-End DNA manufacturing

**Figure 1:**
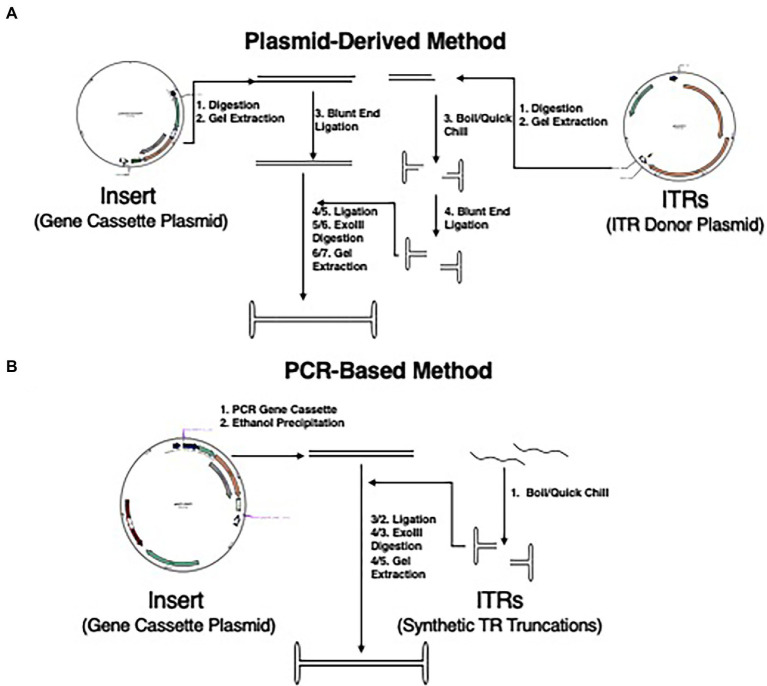
Updated methodology for NE-DNA production. **(A)** Schematic representation of detailed steps of plasmid DNA-derived method of NE-DNA production as described previously (Nahreini et al., 1992). This method was used with plasmids pSub201 and pCBA-EGFP to create NE-WT-ITR-CBA-EGFP. Total steps to completion are 6/7 (Insert/ITR) for NE-DNA generation. **(B)** Schematic representation of the method for the synthesis of NE-DNA production derived from the pAFP-EGFP plasmid and synthetic TR truncations. Total steps to completion are 4/5 (Insert/ITR) for NE-DNA generation.

No-End DNA was generated through two different methods to optimize high yields. The first method generated a NE-DNA construct of bacterial origin, which has been previously described (Figure 1A; Nahreini et al., 1992). Briefly, an AAV plasmid containing a reporter or therapeutic gene cassette was digested by restriction enzymes to yield a linear DNA GOI cassette. The digestions were electrophoresed on 0.8% low-melting point agarose Tris/Borate/EDTA (TBE) gels and the corresponding insert band was gel extracted by phenol:phenol-chloroform two-phase extraction. AAV2 ITRs were excised from pSub201 and extracted using the same method. The ITRs were boiled (100°C for 5 min) and quick-chilled on ice for 20 min. The gel-extracted inserts and ITRs were treated with Klenow polymerase and blunt-end ligated using T4 DNA ligase at a 1:10 insert to ITR ratio at 16°C for 48 h. The reaction mix was incubated at 65°C for 10 min and digested with Exo III nuclease (>1 U/μg) at 37°C for 2 h. The large digestion mixture was electrophoresed on 0.8% TBE low-melting point agarose gel, and the small aliquots were electrophoresed on 1% TAE agarose gels for high resolution visualization. The large digestion mixture was extracted by phenol phenol-chloroform two-phase extraction. The yield of the covalently closed NE-DNA product was measured by nano-drop. This method gave NE-DNA with a mixed pool of sequences containing the wild-type (WT)-ITRs.

A second method was also developed for generating non-bacterial NE-DNA (Figure 1B). The insert was generated using polymerase chain reactions (PCR) of an AAV plasmid containing the desired gene cassette. Phosphorylated primers were selected based upon the flanking regions of the gene cassette in the respective plasmid in between the 5′ promoter sequence or 3′ poly-A tail and the ITRs. The specific sequences were generated by NCBI primer blast and purchased from Eurofins. The polymerase utilized was the high-fidelity Phusion pol (NEB), which generates blunt-ended products. The desired PCR product was verified by gel electrophoresis and isolated by ethanol precipitation. The isolated PCR-generated insert was quantified by nano-drop. AAV2 ITRs and truncations of the AAV2-ITR sequence were designed using a web-based M-fold software from the University of Albany. TR95 was derived from the ITR and was capable of blunt-end folding while retaining the full length RBE. Further blunt-end folding truncations were made from this sequence with TR75 and TR55 maintaining the cruciform structure and having shorter stem lengths, while TR35 was derived from a single hairpin sequence of the cruciform. Synthetic oligonucleotides based on these sequences were purchased from Eurofins in either non- or fully phosphorylated 100uM stock solutions. Phosphorylated ITRs were diluted to 10 μM aliquots in 1× ligase buffer that were boiled for ~5 min and quick-chilled on ice for ~20 min to insure proper folding of hairpin structures, and stored at −20°C.The non-phosphorylated ITRs were diluted to 10 μM using T4 polynucleotide kinase (T4PNK) buffer and incubated at 37°C for 2 h. The ITRs were boiled, quick-chilled, and stored at −20°C.

Isolated insert and properly folded ITRs were ligated together using a 2× Blunt-End ligase in a (1:20) insert to TR ratio to reduce the generation of polymers. For maximal yield, ligations were incubated at RT for 2 h, 48 h at 16°C, and then 48 h at 4°C. The ligation reactions were deactivated and digested with excess Exo III as described above. The digested ligation mixtures were subjected to gel-electrophoresis. NE-DNA was extracted utilizing QIAquick Gel Extraction kit (Qiagen) and sequentially eluted with 20 μl and 10 μl of 37°C nuclease-free H_2_O. Quantification of NE-DNA was done by nano-drop, gel-titering, and qPCR.

### Gel-electrophoresis and imaging/excision

Two different gel electrophoresis protocols were used in this study. The first method was for high resolution visualization for data analysis. These gels were made by melting agarose into Tris-acetate-EDTA generating a 1% (w/v) agarose solution. Ethidium bromide (EtBr) was added to the gel at a concentration of 1 μg/ml for DNA visualization. These gels were run in 1× TAE buffer at 100 mV for 5 min followed by 70 mV for ~45 min. Gels were analyzed utilizing a UVP transilluminator and imaged using Analytica Jena software. The second protocol included gels optimized for DNA extraction. These gels were made using low-melting point agarose in a 0.8% (w/v) melted solution in TBE buffer. EtBr was added at a final concentration of 1 μg/ml. A transilluminator was used to visualize the appropriate NE-DNA monomer band and was excised using a scalpel.

### Gel-titer quantification method

Gene cassette PCR fragment was gel extracted and characterized using nano-drop followed by gel electrophoresis to assess the quantity and quality of the DNA. Once verified for purity, the purified insert was used as a standard with 30, 60, 90, 120, and 150 ng electrophoresed on 1% agarose gels along with NE-DNA of unknown concentration as 2 and 4 μl samples. The gels were run at 100 mV for 5 min followed by 70 mV for 45–60 min. The gels were imaged using a UVP transilluminator and analyzed using the Analytica Jena software VisionWorks. A 1-D analysis of the image yielded an intensity (I-Max) reading of each band at the corresponding kB size. The resulting quantification of the band intensities were exported as an Excel spreadsheet. A standard curve was generated using the known DNA amounts and the resulting I-max values of the purified insert standards. A trendline of the standard curve was generated relating the amount of DNA present in ng to band intensity. The relationship was linear with an *R*^2^ > 0.99 when the intensity imaging was done under exposure times that had no image saturation. The trendline equation was used to calculate the amount of DNA present in the NE-DNA wells and then with the known volume added per well the concentration of the NE-DNA samples was calculated.

### Transfection of DNA into mammalian cells

Transfections of linear DNA, NE-DNA, and plasmids were conducted on the cell lines indicated above. Both calcium phosphate (Current Protocols in Molecular Biology) and Polyethylenimine (PEI) (linear; molecular weight [MW], 40 kDa) (Polysciences, Inc., Warrington, PA, United States) using a ~ 3:1 or ~ 2:1 (w/w) carrier DNA:cargo DNA was utilized to increase the transfection efficiency of non-circularized DNA constructs (Lehner et al., 2013).

Briefly, the transfections were carried out using either 24-well or 12-well dishes seeded with 5×10^4^ or 1×10^5^ cells, respectively, 24 h pre-transfection. At 60–70% confluency the cells were replenished with fresh complete media 1 h before transfection. Calcium-phosphate precipitations were done in 0.1 media volume (MV) with 3.5×10^6^ gc/cell of DNA construct, which translated to ~300 ng per 24-well, ~600 ng per 12-well, and ~ 1.2 μg per 6-well. DNA constructs were mixed with carrier DNA in nuclease free H_2_O. Calcium chloride (2.5 M) was added to the DNA mixture at 0.01 MV and mixed evenly. DNA/CaCl_2_ mixtures were then added to 2×HeBS (0.05 MV) in a dropwise fashion to 2×HeBS (0.28 M NaCl, 0.05 M HEPES (N-2-hydroxyethylpiperazine-N′-2-ethanesulfonic acid), 1.5 mM Na_2_HPO4, pH 7.05). DNA/CaCl_2_/HeBS solution was then mixed thoroughly and allowed to incubate for 20 min to form precipitates. For PEI transfections, DNA constructs were mixed with carrier DNA in PBS and vortexed. PEI aliquots were made in similar volumes, added to the DNA mixture at half volume, and incubated at room temperature for 10–15 min. Newly formed precipitates were evenly distributed dropwise to wells. Cells were incubated at 37°C for 4–6 h and media was aspirated. In the calcium phosphate transfection, the cells were treated with 10% DMSO in complete media at room temperature for 3 min. DMSO media or PEI media was aspirated and cells were washed with 5 MV 100% PBS (1×) and replenished with complete media.

### Western blot analysis

Western blot assays were performed as previously described (Dai and Siemann, 2010). The following antibodies were used: monoclonal anti-hF.IX (1:1,000 dilution; LSBio, Inc., Seattle, WA, United States); monoclonal anti-actin (1:1000 dilution; Santa Cruz Biotech., Inc., Dallas, TX, United States); horseradish peroxidase-conjugated secondary antibodies (1,1,000 dilution; Santa Cruz Biotech., Inc., Dallas, TX, United States).

### Spectrofluorometric quantification and analysis

Fluorescence quantification was done using a BioTek Synergy HTX Multi-Mode Microplate Reader. Transfected cells in 6-well plates were recorded for fluorescence after culture media was aspirated and replaced with 100% PBS. Resulting quantification was recorded as arbitrary fluorescent units (AFU) and were exported to a Microsoft Excel sheet for analysis. The mean average of the mock-transfected wells was subtracted from the means of the respective EGFP-carrying constructs. Bar graphs and Standard Deviations were made based off these mock-subtracted results.

### Fluorescence imaging and analysis

Cell fluorescence imaging was done 3 days post-transfection and weekly thereafter with a Leica fluorescence microscope. At 50× magnification, each well was imaged with 8 images per well for 12 well plates and 13 images for 6 well plates. Initial imaging calibration was done with mock- transfected cells and then applied to all other wells. All subsequent transfection imaging was done under the same parameters allowing for a direct comparison between time points. Image analysis was done using ImageJ and standardized by a macro script that controlled for all the established ImageJ parameters. This ImageJ macro was run on all the images from each timepoint. The quantification of the images was then analyzed in Excel with bar graphs being generated for each time point.

### Isolation of low molecular weight DNA

Extra-chromosomal DNA was extracted at various timepoints post-transfection using Hirt’s DNA isolation buffer (10 mM Tris, 10 mM EDTA, 0.6% SDS, and pH 8.0) described by Hirt (1967). At each timepoint, following imaging, cells in each well were trypsinized and split into two aliquots. One aliquot was kept for continued culturing and the other aliquot of cells were subjected to Hirt’s DNA isolation. Cells were pelleted at 3,000 rpm for 5 min and most of the supernatant was removed except 100 μl. Cells were resuspended by pipet and 400 μl of warmed fully dissolved Hirt’s solution was added to each sample and inverted 4–6×. Supersaturated 2.5 M NaCl was then added to each sample (200 μl) and inverted 4–6×. Samples were then incubated in 4°C for 24–72 h. Samples were the centrifuged at full speed (13,000 rpm at 4°C). The supernatant was sequestered from pelleted cell debris into a new tube where one volume of phenol-chloroform (1:1) ~600 μl was added to each sample and inverted (>10×). The samples were centrifuged at full speed for 20 min generating two phases. The top aqueous phase was aliquoted to another tube and subjected to a repeat two-phase extraction with 100% chloroform solution. The twice extracted aqueous layer was precipitated with one volume (~600 μl) of iso-propanol and 20 μl of sodium acetate. Samples were incubated at −20°C overnight and pelleted at 13,000 rpm centrifugation for 20 min. Samples were washed once with 70% ethanol and the pellets were dissolved in 50 μl of nuclease-free H_2_O.

### Half-life analysis

An exponential decay analysis was performed to generate a gene-expression half-life of each construct. This involved constructing a scatter-plot of the gene-expression profile each DNA construct over the time of the study with various time-point values. An exponential trendline of each DNA construct’s overall gene-expression regression gave an exponential decay equation. Dividing the natural log of 0.5 by lambda yielded the half-life in days of each DNA construct. A relational analysis was performed of the gene-expression half-life results by using linear DNA as a standard and its relation to the other DNA constructs (plasmid and NE-DNA).

### Data analysis and *t*-test selection

Statistical methodology included an analysis of data to generate to generate SD and *p*-values to determine significance among experimental groups. Results were shown as bar graph with means ± SD. To generate *p*-values a specific *t*-test was done depending on the relationship between the data groups. If the means of the two groups were within 1 SD of each other from either perspective, then it was deduced that there is a low probability that one of groups has a larger or smaller value than the other. In this circumstance a two-tail *t*-test was implemented, which generates a *p*-value that assumes both instances that there is a chance of group A being larger than group B and *vice-versa*. If the means of the two groups were not within 1 ± SD of each other, then it was deduced that there is a high probability that one group is larger than the other. In this circumstance a one-tail *t*-test was implemented, which generates a *p*-value that assumes that group A is larger than group B or *vice-versa*. Further *t*-test specificity was done using an F-test to probe if the groups have equal or unequal variance. Thus, four different iterations of *t*-test were implemented using deductive statistical methodology to generate fully characterizing *p*-values: two-tailed *t*-tests with un-equal or equal variance and one-tailed *t*-tests with un-equal or equal variance.

## Results

### Optimization of the NE-DNA manufacturing protocol

The different methodologies of creating NE-DNA were compared regarding time, resources, and DNA yield. The plasmid derived method (Figure 1A) could create efficient Exo-III resistant NE-DNA constructs, but the yield was very low with ~10% of the initial plasmid-derived insert being properly ligated. Scaling up upstream production with this method would lead to bottlenecks in plasmid purification, which is a resource heavy, time-consuming endeavor. Additionally, the epigenetic profile of bacterial originated NE-DNA constructs has been found to be a potential hindrance in long-term gene expression in eukaryotes (Nafissi et al., 2014). The PCR-derived method (Figure 1B) was capable of GOI insert generation in much shorter time-scales (~5 h vs. ~48 h) and was devoid of epigenetic alterations. The polymerase used was high fidelity Phusion polymerase capable of blunt-end product generation. This removed the need of blunt-ending the insert product before ligation as needed in the plasmid-derived method. The introduction of synthetic TR oligonucleotides reduced the need of digestion and gel-extraction from plasmids, as well as introduced an effective strategy to do a comparative analysis of different TR truncations. The ligation ratio of 1:10 (insert:ITR) was found to be effective in generating exonuclease-resistant NE-DNA, but multiple polymer species were also generated. Increasing the ratio to 1:20 was found to greatly reduce the number of NE-DNA polymers with the predominant construct being the desired monomer. Ligation incubations were tried at various temperatures and times yielding an optimized incubation protocol of 2 h at RT, 48 h at 16°C, and ~48 h at 4°C.

### Transgene expression longevity of WT-ITR-125-NE-DNA compared with linear construct

**Figure 2:**
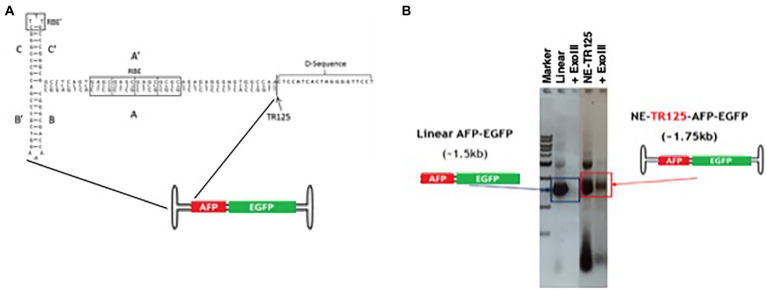
Characterization of wt-125-NE-DNA derived from ITR. **(A)** Schematic of wt-ITR-125-NE-DNA with its conserved ITR domains. **(B)** Agarose gel image of wt-ITR-125-NE-DNA ligations constructed from the ITR before and after extensive ExoIII digestion (>10 IU/μg of DNA). Open-ended, linear DNA was used as a control. Digestions were incubated at 37°C for 2 h.

To determine if NE-DNA has increased longevity over linear DNA, a NE-DNA construct was made using a synthetic TR oligonucleotide derived from the WT-ITR sequence. This 125 nucleotide (nt) oligo was a D-sequence removed variant of the known 145 nt sequence, which was covalently ligated to an insert (AFP-EGFP) (Figure 2A). NE-WT-TR125-AFP-EGFP (NE-TR125) was validated for exonuclease resistance (Figure 2B) and was compared with open-ended, linear PCR fragment (linear DNA), plasmid (pAFP-EGFP), and mock by calcium-phosphate transfection in Huh-7 cells.

**Figure 3:**
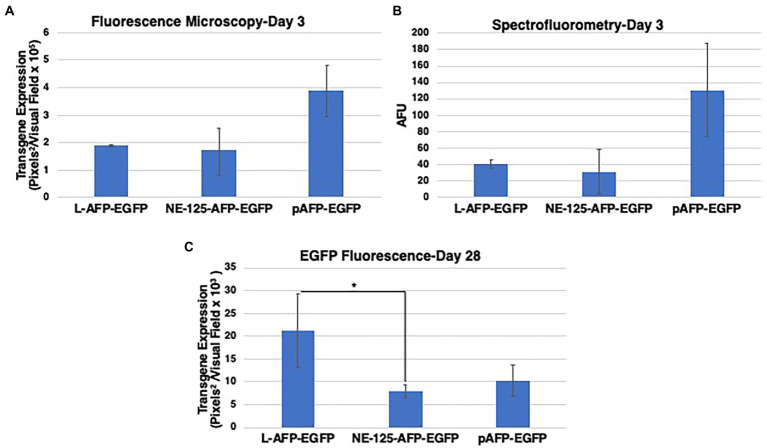
Data validation and comparative analysis of EGFP fluorescence from NE-TR125-AFP-EGFP, Linear open-end (L) AFP-EGFP, and circular Plasmid pAFP-EGFP. Two techniques were used at Day 3 to determine the best quantifiable methodology. **(A)** Quantified fluorescence imaging data using fluorescence microscopy and ImageJ analysis of EGFP transgene expression at 3 days post-transfection. **(B)** Quantified fluorescence as measured by spectrophotometry as artificial fluorescent units (AFU). Fluorescence imaging data were collected at day 28 **(C)** post-transfection and quantified by ImageJ. Two-tailed unequal variance *t*-test utilized to generate *p*-values. *Y*-axis of each bar graph are adjusted to the respective data. *p < 0.05. Bar graphs represent the mean fluorescence of the triplicates with ±1SD error bars.

EGFP fluorescence was measured at Day 3 with two methodologies to validate imaging techniques, fluorescence imaging and spectrophotometer. Transgene expression levels measured individually by both techniques were able to come to a similar consensus (Figures 3A,B). Fluorescence imaging proved to have lower data variability than spectrophotometer readings. The study was continued with fluorescent imaging analysis at Day 7 and weekly thereafter till Day 28.

The fluorescence data illuminated that at Day 3 there was no difference between linear DNA and NE-TR125-DNA with plasmid having a roughly twofold increase in fluorescence comparatively (Figure 3A). Over the subsequent weeks NE-TR125-DNA fluorescence decreased in relation to linear DNA with Day 28 indicating a threefold smaller level of transgene expression as compared to linear-DNA. Plasmid DNA saw a faster systematic decrease in transgene expression as compared to the other constructs with a steep drop off from Day 14 to Day 21. By Day 28 there was no difference between plasmid and NE-DNA (Figure 3C).

### NE-DNA constructs derived from ITR truncations outperform the linear DNA

**Figure 4:**
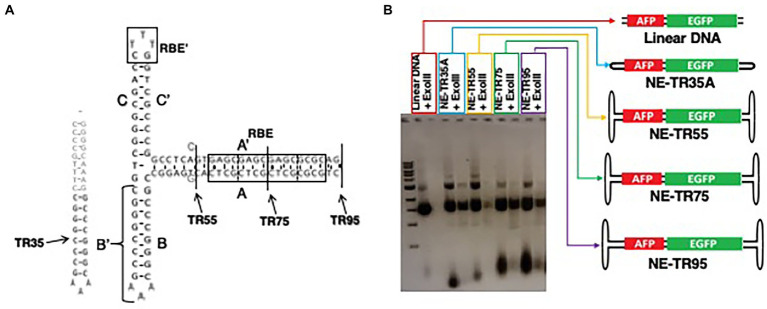
Characterization of NE-DNA derived from ITR truncations. **(A)** Schematic of ITR truncations with their conserved ITR domains. Gray sequences were added/substituted to increase the stability of blunt-end folded confirmations. **(B)** Agarose gel image of NE-AFP-EGFP ligations constructed from the scITR truncations before and after extensive ExoIII digestion (>10 IU/μg of DNA). Open-ended, linear DNA was used as a control.

To determine if the ITR sequence may affect the longevity profile of NE-DNA, constructs were designed and made using the AAV2 ITR sequence as the template. A total of 4 TR truncations were made using this template including TR-35 (a single hairpin structure with B/B′ domain plus an additional synthetic 18 nt sequence for increased stability), TR55 (short A/A’ stem cruciform structure void of the RBE domain), TR75 (medium A/A’ stem cruciform structure with half an RBE domain), and TR95 (long stem cruciform structure with a full RBE domain) (Figure 4A). All four were blunt-end ligated to the DNA insert AFP-EGFP as mentioned in the materials and methods section. The NE-DNA construct digestions to verify exonuclease resistance as comparted to linear DNA (Figure 4B).

**Figure 5:**
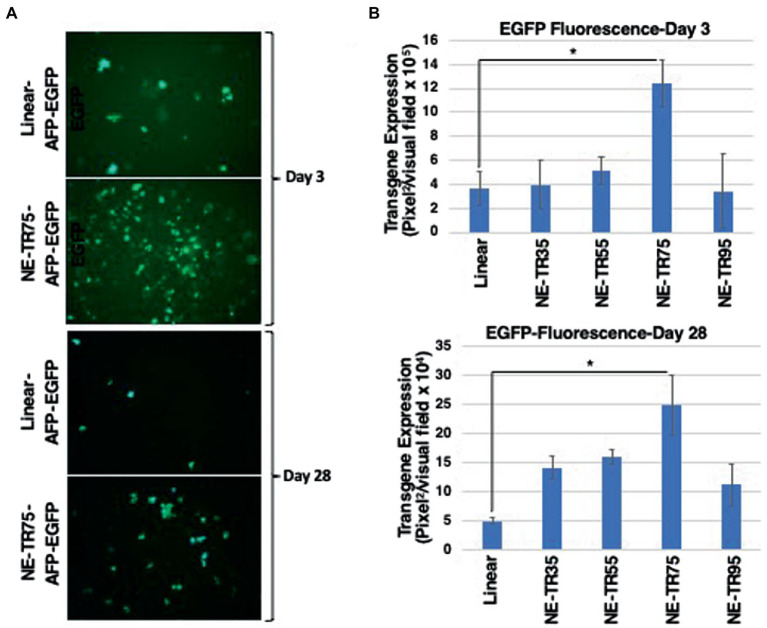
Evaluation of transgene expression from NE-TR-AFP-EGFP constructs. The bar graphs correlate to measured fluorescence through ImageJ with ±1SD error bars. **(A)** Images represent the closest captured image that corresponds to the median fluorescence of the constructs with the highest and lowest transgene expression. **(B)** The GFP transgene expression of the constructs 3 days and 28 days post-transfection in Huh7 cells. *Y*-axis of each bar graph are adjusted to the respective data. A one-tail *t*-test with un-equal variance was used to generate *p*-values. *p < 0.05.

All NE-DNA constructs were found to have improved exonuclease resistance over linear DNA, but one construct (NE-TR55-DNA) did not show the same level of resistance as the other NE-DNA constructs. These constructs, in addition to the control linear DNA, were CaPO_4_ transfected into Huh-7 cells and monitored in the same manner as the previous NE-WT-TR125 experiment. GFP imaging was done a Day 3 and Day 7 with weekly observations thereafter. At Day 3 NE-TR35, NE-TR55, and NE-TR95 had no significant difference amongst themselves or the linear DNA control, but NE-TR75 was found to have a substantially higher (threefold) transgene expression level than linear DNA (*p*-value<0.05). As time progressed, this relationship was maintained through Day 7-Day 28 at which time NE-75 was found to have a fivefold increase over linear DNA (*p*-value <0.05) (Figure 5) Also at Day 28, the other NE-DNA constructs (NE-TR35 and NE-TR55) were found to have a ~ threefold larger transgene expression levels than linear DNA, while NE-TR95 was found to have a ~ twofold higher expression level than linear DNA (not significant).

### Optimized NE-DNA mediates increased expression longevity and DNA stability

**Figure 6:**
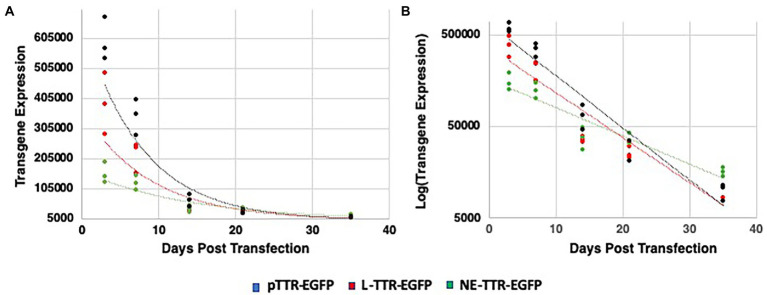
Decay quantification and trend analysis of transgene expression from pTTR-EGFP, linear TTR-EGFP, and NE-TR75-TTR-EGFP over a 35-day interval following transfection. Scatterplots were generated for each construct with ImageJ transgene expression data vs. days post-transfection and a best-fit analysis was conducted to generate a trendline equation. **(A)** Scatter-plot analysis yielded an exponential decay trend-line, which was then linearized logarithmically in panel **(B)** to generate a rate of decay equation used to conduct a transgene half-life analysis.

Quantitative analysis of transgene regression was done to verify if optimized NE-DNA design derived from the AAV2-ITR sequence (NE-TR75) was applicable to a different gene cassette. A NE-TR75 construct was made using a TTR-EGFP PCR fragment insert, which was again verified for its exonuclease reactivity before application *in vitro*. Following the same methodology as before, this construct was transfected into Huh-7 cells alongside controls (linear TTR-EGFP and pTTR-EGFP). These cells were followed for 5 weeks with GFP imaging being done at Day 3 and Day 7, weekly thereafter. ImageJ data from all time points were analyzed *via* Excel spreadsheet generating scatterplots (Days Post-Transfection vs. Transgene Expression) for each DNA construct. A gene expression half-life for each DNA construct was generated using a best-fit trendline analysis. Out of the three trendlines assessed (linear, logarithmic, and exponential) the exponential trendline had the best R-squared values for all constructs indicating the timed regression of transgene expression followed an exponential relationship, thus allowing for an exponential decay analysis (Figure 6A) Linear DNA, NE-DNA, and Plasmid exhibited a half-life of 6.1, 9.8, and 5.3 days, respectively, (Figure 6B).

**Figure 7:**
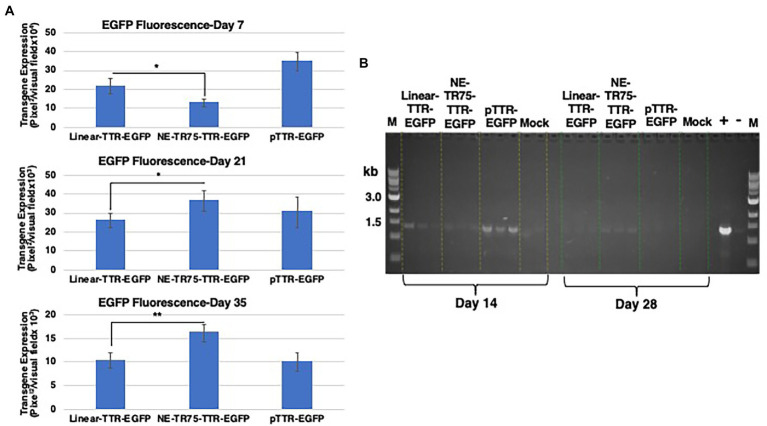
Comparative analysis of transgene expression from linear TTR-EGFP, NE-TR75-TTR-EGFP, and plasmid pTTR-EGFP DNAs **(A)** at day 7, (top panel) day 21 (middle panel), and day 35 (bottom panel) post-transfection in Huh7 cells. Day 7 *p*-value was calculated using a one-tail, unequal variance *t*-test. Day 21 and day 35 *p*-values were calculated using a one-tail, equal variance, *t*-test. *Y*-axis of each bar graph are adjusted to the respective data. *p < 0.05, **p < 0.01. **(B)** Gel electrophoresis of low mol. wt. DNA of PCR-amplified products. Approximately, 1 μg of Hirt’s DNA isolated at indicated time points were used for a 30 cycle PCR analysis. Equivalent amounts were electrophoresed on a 1% agarose gel and visualized under UV light. Positive (a band at ~1.5 kb) and negative controls are shown on right. M = markers.

A factor of decay was calculated with the standard being the linear DNA half-life, which found that NE-DNA had a ~ 1.6-fold longer half-life than that of Linear. This can be seen in the bar graph analysis of transgene expression profiles as well with NE-DNA initially at day 7 under-performing linear DNA (*p*-value <0.05), but by day 21 the trend had reversed with NE-DNA having a ~ 1.4-fold higher transgene expression than linear DNA. This relationship increased to a ~ 1.6-fold difference by day 35 (Figure 7A).

Semi-quantitative analysis of expression cassette DNA was performed as follows. Low mol. wt. DNA samples were isolated at each time point after Day 7 to assess whether the presence of extra-chromosomal constructs correlated with the observed transgene expression levels. This was done by splitting the cells in each well into two groups, half for continued culturing and the other half for Hirt’s solution DNA extraction. Extra-chromosomal DNA was extracted as detailed in the Materials and Methods. These extractions were stored in −20°C until all time points were obtained. PCR-analysis of the low mol. wt. DNA was done at timepoints Day 14 and Day 35. The amplicon was the full TTR-EGFP ~1.5 kb insert, which was then ran on a 1% agarose gel (Figure 7B). At Day 14, the strongest bands were those of the plasmid group followed by the linear DNA group and then the NE-DNA group. This correlates to what was observed at the protein level with the transgene expression at Day 14 indicating roughly the same relationship. At Day 35, all bands were of lower intensity than the previous time point, yet there was an observable difference between the linear DNA group and the NE-DNA group. Analytica Jena Vision Works software was used to quantifiably determine respective band intensity, which showed the NE-DNA group was ~1.2-fold higher than that of the linear DNA group. This correlates with Day 35 ImageJ transgene expression data showing higher fluorescence from the NE-DNA group. Thus, it can be deduced that the presence of the DNA constructs correlated with their respective gene expression profiles at the various timepoints.

### Optimized NE-TTR-hF.IX construct exhibited higher hF.IX transgene expression than L-TTR-hF.IX 28 days post-transfection

**Figure 8:**
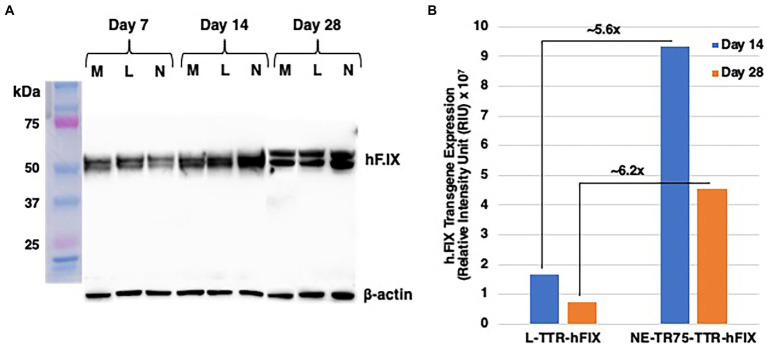
Western blot analysis of comparative hF.IX expression from L-TTR-hF.IX and NE-TR75-TTR-h.FIX. HepG2 cells plated in a 10 cm dish were either mock-transfected or transfected with NE-TR75 TTR-hF.IX or Linear-TTR-hF.IX DNA. **(A)** Equal concentrations of each protein were analyzed on Western blots using monoclonal anti- hF.IX and anti-β-actin antibodies as described under Materials and Methods. **(B)** Quantitation of the data from **(A)**. M, mock; L, linear DNA; N, NE-DNA.

To verify whether optimized NE-DNA could be utilized in a therapeutic model for hemophilia, a NE-TTR-hF.IX construct was generated to test its applicability in delivering a therapeutic gene. This construct was transfected into HepG2 cells, which are known for their ability to produce and secrete hF.IX (De La Sale et al., 1985; Gordon et al., 1993), along with Linear-TTR-hF.IX and a mock transfection as a negative control. Whole cell lysates (WCL) and low molecular DNA were isolated at day 7, day 14, and day 28 post-transfection. WCLs were subjected to western blot analysis incubated with hF.IX antibody and beta-actin antibody as a control. At Day 7 all the constructs exhibited similar hF.IX protein levels, however by day 14 NE-TTR-hF.IX construct surpassed L-TTR-hF.IX in hF.IX expression. This trend was sustained to day 28 indicating that NE-TTR-hF.IX presented a longer transgene expression profile than that of L-TTR-hF.IX (Figure 8A).

A further quantitative analysis was conducted on the western blot image, which involved subtracting mock hF.IX expression from L-TTR-hF.IX and NE-TTR-hF.IX, this was done to accommodate for the native background hF.IX expression in Hepg2 cells (Figure 8B). Once adjusted for mock the western blot quantitation indicated a roughly ~5.6-fold increase in hF.IX expression from NE-TTR-hF.IX in relation to L-TTR-hF.IX at day 14. At day 28 this relationship not only sustained but increased to ~6.2-fold higher hF.IX expression from NE-TTR-hF.IX compared to L-TTR-hF.IX.

## Discussion

It is clear that AAV vector-mediated gene therapy of hemophilia in children will require repeat dosing since up until ~10–12 years of age, the liver is still growing, and hepatocytes are undergoing cell divisions, and due to the episomal nature of the vector genome, resulting in dilution and loss of the therapeutic gene over time. However, repeat vector administrations are not possible, given the development of NAbs to AAV capsid proteins following the first dosing. Although a number of promising approaches to modulate the host humoral response to AAV vectors, which include transient B-cell ablation (Corti et al., 2014, 2015), enzymatic degradation of pre-existing IgGs using IdeS (Leborgne et al., 2020) and IdeZ (Elmore et al., 2020), and AAV vector-mediated downregulation of the major histocompatibility class II (MHC-II) gene expression (Kwon et al., 2020), are currently being evaluated in pre-clinical models, it is also clear that there is need for the development of a non-viral vector capable of evading the host immune surveillance, as well as repeated delivery therapeutic genes.

Plasmid DNA has been used to this end by satisfying the first two characteristics, but because of their bacterial epigenetic and sequence profile, its clearance by intracellular mechanisms greatly decreases its longevity *in vivo*. Linear DNA, derived from PCR, containing a gene of interest (GOI) devoid of any bacterial epigenetic factors has been shown to have increased longevity and bioavailability (Lehner et al., 2013). Capitalizing on this advantage, we postulated that the utilization of the previously described construct NE DNA, a DNA-based construct with the addition of AAV2 ITR sequences on either side of a linear GOI construct, should exhibit augmented longevity compared with the linear GOI construct alone.

This study demonstrates that an optimized novel, AAV genome-based, covalently closed TR75-DNA construct (optNE-DNA) can increase transgene longevity in comparison to both linear open-ended DNA and plasmid DNA. The design was proven to be modular with different gene cassettes utilized indicating the same relative transgene expression longevity relationship regardless of promoter-gene combination. This NE DNA (NE-DNA) was previously found to be exonuclease-resistant, capable of expressing its cargo in transfected cells, integrating into the genome in the presence of the AAV Rep protein, being replicated in the presence of AAV Rep and adenovirus 2 (Ad2) proteins (Nahreini et al., 1992). With its increased stability due to the addition of flanking ITRs and its ability to express an inserted transgene, NE-DNA was an excellent candidate to test for longevity. In the present studies, we further improved the manufacturing protocol of NE-DNA to a synthetic DNA generated by PCR and optimized its design to increase DNA stability and transgene expression longevity over standard DNA-based vectors. A roughly ~1.6-fold increase in protein-level transgene expression half-life (days) and increased DNA-level persistence, as compared to open-ended linear and plasmid DNA, was observed with an optimized NE-DNA (NE-TR75-DNA) construct generated from a truncation of the ITR sequence. This NE-TR75-DNA design also yielded longer therapeutic hF.IX expression than that of Linear DNA at both Day 14 and Day 28 with a mock-adjusted increase of ~sixfold.

Taken together, these data suggest that the transgene expression from, and the DNA stability of, the NE-TR75-DNA (opt-NE-DNA) were significantly higher than those of other NE-DNA constructs as well as of both linear and plasmid DNAs. We offer a plausible mechanism underlying these observations as follows. The first construct made was the previously mentioned NE-AFP-EGFP-TR125 (NE-125), which was compared with linear and plasmid DNA controls in Huh7 cells. The results of this study found that linear DNA, and not NE-125, had the most sustained gene expression profile with no significant difference between plasmid and NE-125 transgene expression. This was understood to be the possible result of the ITR sequence itself as it was previously found that WT-ITR containing constructs have repressed gene expression as opposed to those without ITR sequences. This is because AAV ITRs can bind to the double-strand break pathway protein ataxia-telangiectasia mutated (ATM), which can repress gene expression directly (Bijlani et al., 2022). Additionally, the activation of the ATM due to the presence of ITRs can induce cell cycle arrest and apoptosis (Pusapati et al., 2006). A full length ITR may have increased association with ATM limiting the amount of transcription.

One consideration is the efficiency of TR75 over the other TR truncations, which is thought to be the consequence of overall length of structure or the presence/absence of a specific sequence domain. The correlation between longer TR truncation length and increased transgene expression levels seems to be applicable with the shorter three TRs (TR35, TR55, and TR75). Figure 5B shows this correlation with a positive relationship between transgene expression and TR length within the three TR truncations mentioned. This could be the result of increased DNA protection from nucleases due to longer TR sequences combined with less structure hindrance with longer TRs allowing for larger de-annealed gene expression complexes to form than the tighter, shorter TRs. Though applicable with the smaller three truncations, this hypothesis fails to be applicable with the longest TR: TR95. Following the trend of this hypothesis, TR95 should have the highest level of transgene expression, yet it is one of the poorest performing TRs out of the group.

To inquire as to what may be the reasoning for this drastic difference between TR75 and TR95, a direct comparison of the sequences and structures of both are shown in Figure 4A. The main notable difference is that TR95 has a full intact 16 bp RBE (rep-binding element), while in TR75 this site is truncated in half. This RBE site has been implicated in various different utilizations from proper AAV genomic resolution to rep-dependent site-specific AAV genome integration into AAVS1 on chromosome 19 (Xu et al., 2006). Rep-protein binding to the RBE has been found to repress transgene expression and there have been Rep homologs in other viruses including human-herpesvirus 6 (HHV-6) that do the same (Thomson et al., 1994; Pereira et al., 1997). Although there have been no known human protein homologs thus far that explicitly have Rep homology in binding to RBEs, it has been estimated that there could be as many as 2×10^5^ sites found in the human genome that have homology with the 8-bp minimal RBE domain (GAGYGAGC) (Young Jr. et al., 2000). To determine if there are any known human transcription factors that bind to the AAV2-RBE the ITR sequence from the beginning of the RBE to the end of the D-sequence was analyzed by the transcription factor motif database, footprintDB. The inquiry found homology between the distal 8 nucleotides of the RBE and the binding domain of E2F2 (GCGCGCGC). E2F2 is a well characterized transcription factor involved in cell cycle progression through both repressor and activator modalities (Müller et al., 2001). Ablation of the E2F family proteins in mouse models show they have an association with tissue homeostasis and tumor genesis (DeGregori and Johnson, 2006). It is quite possible that in the case of the AAV-ITR, the stimulation of the DSB response by the ITR could cause E2F2 to serve a repressor role to reduce activation of genes proximal to DSBs. TR95 carries this homology to the E2F2 binding domain (GCGCGCGC) whereas TR75 does not. Thus, the transgene expression difference between TR75 and TR95 may be attributable to a truncated vs. full intact RBE domain, while the difference among the smaller three TR truncations (TR35, TR55, and TR75) may be the result of a positive correlation between the length of the TR structure and increased DNA stability.

The direct application of NE-TR75-DNA (optNE-DNA) to hemophilia in children resides on its longevity profile. As previously mentioned, the only viable option for children with hemophilia is prophylactic factor infusions on a very stringent repeated dosing schedule because of the relatively short half-life of the proteins. The goal of optNE-DNA is to extend the therapeutic half-life of a single dose by providing a stable DNA-based therapy coding for the factor protein that is capable of longer serum factor levels than that of prophylactic factor infusions and other non-viral DNA-based factor gene delivery mechanisms (i.e., PCR fragment, plasmids), while still maintaining repeat dosing capabilities that correct for episomal dilution of the DNA in a child’s growing liver. The optimization of NE-DNA’s manufacturing process and design are the first steps towards this endeavor and has thus far proven optNE-DNA to be increasingly stable at the DNA level and capable of longer transgene expression at the protein level than the compared DNA-based methodologies holding much promise as a potential therapeutic for childhood hemophilia.

The implications of this study extend beyond the treatment of childhood hemophilia B. Theoretically, if opt-NE-DNA is further proven to be competent of increased transgene expression longevity to a therapeutic end and is capable of robust expression *in vivo* when packaged in conjugated liposomes, then the proof-of-concept of this technique would be validated, and this technology could be applied to a multitude of mono-genetic disorders. The modulation at that point could be two pronged with the GOI in the opt-NE-DNA construct and tissue-targeting with the selective conjugation of the liposome. With no presently defined GOI size restriction with optNE-DNA, this therapy could be implemented in those disease models that are outside the realm of standard AAV’s packaging capacity of 4.7 kb. Theoretically, full-length F.VIII could be ligated into a single optNE-DNA construct and provide disease remediation for those that suffer from hemophilia A. This is currently an issue for standard AAV therapy, even when using the truncated B-domain deleted hF.VIII gene, because the length of the gene cassette (~5.0 kb) is larger than the carry capacity of AAV (~4.7 kb) (Roberts et al., 2011). OptNE-DNA carrying hF.VIII gene could be packaged into the liver-targeting liposomes and used in adult patients. The size capacity could also allow for two genes to be package into a single optNE-DNA molecule. Dual gene approaches have been implemented in various disease models including non-small cell lung cancer (NSCLC) in which it was found that the addition of plasmids coding for tumor suppressor genes p53 and Fus1 reduced the number of tumors by 70–80% in murine models over a period of 48 h (Deng et al., 2007). Ligating both these genes into optNE-DNA and packaging them into NSCLC-targeting liposomes could robustly translate these findings into human models with little off-targeting and sustained expression. Both approaches would be further augmented by the option of repeat dosing, which is currently not possible with standard AAV vector-mediated gene therapy.

Additional studies are warranted to assess the therapeutic potential of the opt-NE-DNA construct in an animal model *in vivo*. Hydrodynamic delivery of plasmid DNA in murine models has been used to transfect hepatocytes and could be utilized to determine if optNE-DNA’s transgene longevity profile is maintained murine hemophilia models with repeat administrations (Bonamassa et al., 2011). Additionally, to improve the bio-distribution profile of the naked DNA construct, we postulate that opt-NE-DNA could be packaged into liver-targeting liposomes. Studies have shown that conjugated liposomes are not only capable of evading NAbs, but also can be very efficient in cargo delivery and are modular to various tissue specific cell lines. Conjugations of liver-specific polymers in liposome formulations have been developed for treating HCC tumors over the years with increasing hepatic transfection efficiency, longer retention times, and decreased cytotoxicity compared with non-conjugated liposomes *in vivo*. This strategy has been applied to gene therapy with galactosylated (GAL) conjugated liposomes carrying siRNA as a therapy for HCC tumors, which was capable of robust transfection of Huh7 cells while relatively low transfection of A549 cells, a lung epithelial carcinoma (Oh et al., 2016). Delivery of opt-NE-DNA carrying the hF.IX gene by employing a packaging vector like GAL-liposomes may hold promise for childhood hemophilia patients if factor levels >5% can be established to perturb severe symptoms and maintained with fewer administrations than regular prophylactic factor infusions. Since there is an absence of immunogenic viral proteins that may illicit an immune response this methodology, the therapeutic application of this approach could be immense and potentially much more advantageous than standard-of-care prophylactic factor infusions and standard AAV-based gene therapy approaches for children. It is also tempting to speculate that this approach could be a promising addition to the currently available approaches for the potential gene therapy of a wide variety of human liver diseases.

## Data availability statement

The original contributions presented in the study are included in the article/supplementary material, further inquiries can be directed to the corresponding author.

## Author contributions

JS and KQ performed the experiments. JS, KQ, and AS analyzed the data. AS conceived of the idea. JS and AS wrote the manuscript. All authors contributed to the article and approved the submitted version.

## Funding

This research was supported in part by a 2021 Global Hemophilia ASPIRE grant from Pfizer; a Public Health Service grant R01GM-119186 from the National Institutes of Health, and support from the Kitzman Foundation to AS.

## Conflict of interest

AS is a cofounder of, and holds equity in, Lacerta Therapeutics. He is also a paid consultant to Passage Bio and AgeX Therapeutics. JS, KQ, and AS are inventors on several issued/filed patents on recombinant AAV vectors that have been/are being licensed to various gene therapy companies.

## Publisher’s note

All claims expressed in this article are solely those of the authors and do not necessarily represent those of their affiliated organizations, or those of the publisher, the editors and the reviewers. Any product that may be evaluated in this article, or claim that may be made by its manufacturer, is not guaranteed or endorsed by the publisher.
